# Titanium Dioxide Nanoparticle-Biomolecule Interactions Influence Oral Absorption

**DOI:** 10.3390/nano6120225

**Published:** 2016-11-29

**Authors:** Mi-Rae Jo, Jin Yu, Hyoung-Jun Kim, Jae Ho Song, Kyoung-Min Kim, Jae-Min Oh, Soo-Jin Choi

**Affiliations:** 1 Division of Applied Food System, Major of Food Science and Technology, Seoul Women’s University, Seoul 01797, Korea; mirae8651@naver.com (M.-R.J.); ky5031@swu.ac.kr (J.Y.); 2 Department of Chemistry and Medical Chemistry, Yonsei University, Wonju, Gangwondo 26493, Korea; hjun.kim@yonsei.ac.kr (H.-J.K.); dielion@naver.com (J.H.S.); pttkkm@gmail.com (K.-M.K.); 3 Seoul Institute, National Forensic Service, 139, Jiyang-ro, Yangcheon-gu, Seoul 08036, Korea

**Keywords:** titanium dioxide, interaction, biomolecule, oral absorption, intestinal transport

## Abstract

Titanium dioxide (TiO_2_) nanoparticles (NPs) have been widely applied in various industrial fields, such as electronics, packaging, food, and cosmetics. Accordingly, concerns about the potential toxicity of TiO_2_ NPs have increased. In order to comprehend their in vivo behavior and potential toxicity, we must evaluate the interactions between TiO_2_ NPs and biomolecules, which can alter the physicochemical properties and the fate of NPs under physiological conditions. In the present study, in vivo solubility, oral absorption, tissue distribution, and excretion kinetics of food grade TiO_2_ (f-TiO_2_) NPs were evaluated following a single-dose oral administration to rats and were compared to those of general grade TiO_2_ (g-TiO_2_) NPs. The effect of the interactions between the TiO_2_ NPs and biomolecules, such as glucose and albumin, on oral absorption was also investigated, with the aim of determining the surface interactions between them. The intestinal transport pathway was also assessed using 3-dimensional culture systems. The results demonstrate that slightly higher oral absorption of f-TiO_2_ NPs compared to g-TiO_2_ NPs could be related to their intestinal transport mechanism by microfold (M) cells, however, most of the NPs were eliminated through the feces. Moreover, the biokinetics of f-TiO_2_ NPs was highly dependent on their interaction with biomolecules, and the dispersibility was affected by modified surface chemistry.

## 1. Introduction

Since the 21st century, nanotechnology has expanded its applicability to various industrial fields including electronics [1], chemical processes [2], medicines [3], and other bio-related fields [4]. Among various nanoparticles (NPs), titanium dioxide (TiO_2_) has attracted tremendous interest as it has attractive physicochemical properties such as semiconducting characteristics [5], photocatalytic effects [6], a high refractive index [7], etc. Indeed, human beings are increasingly being exposed to TiO_2_ NPs due to their extensive applications as food whitening additives [8,9] or sunscreen agents [10]. Diverse particle sizes and unexpected semiconducting properties of TiO_2_ NPs have led to increasing arguments regarding their toxicity. In the early stages of NP toxicity studies, the cytotoxicity of TiO_2_ NPs was studied in terms of both their high specific surface area and semiconducting properties. Donaldson et al. reported that small TiO_2_ particles (~20 nm) showed more plasmid breakage than larger TiO_2_ (~500 nm), when they were incubated with plasmid DNA [11]. They also suggested that the surface characteristics of TiO_2_ NPs were crucial to generate free radicals, which mediates DNA destruction. It was also well demonstrated that the semiconducting properties of TiO_2_ NPs could affect cytotoxicity. Uchino et al. reported that Chinese hamster ovary (CHO) cells incubated with Aeroxide^®^ P25 (20 nm sized TiO_2_ NPs produced by Evonik Industries) showed significantly reduced viability after ultraviolet ray irradiation [12]. Zhang et al. also reported that the cell viability of human colon carcinoma LS147T cells significantly decreased to 20% by simultaneous ultraviolet (UV) irradiation and TiO_2_ NP treatment, while only NP-treated cells exhibited more than 90% of their original viability [13].

Despite several studies on the potential cytotoxicity of TiO_2_ NPs, in vivo studies revealed their low oral toxicity. An in vivo study showed that orally administered TiO_2_ NPs (5 mg/kg body) did not induce significant translocation of TiO_2_ into rat organs, suggesting their potential excretion through the feces [14]. Warheit et al. reported that acute oral toxicity of TiO_2_ NPs was very low and even skin irritation and pulmonary toxicity were not significant [15]. Warheit et al. also studied acute and subacute oral toxicity of several types of pigment grade TiO_2_ NPs, showing no remarkable oral toxicity caused in rats [16].

In order to predict the potential in vivo toxicity of TiO_2_ NPs, it should be primarily understood how NPs behave in biomatrices, which contain a variety of chemical species spanning from small molecules and electrolytes to macromolecules such as proteins. Several studies have described that NPs actively interact with various molecules, leading to modification of their colloidal properties such as dispersibility and agglomeration [17,18,19]; their surface chemistry can be also modified in the presence of biomolecules [20,21]. Once NPs are exposed to biological media, they can form protein corona, and this NP-protein complexation can determine their biological fate such as cellular recognition [22]. Another important factor in understanding the in vivo behaviors and toxicity potential of orally administered NPs is the possibility of NP transport across the gastrointestinal barriers. In vitro studies on human intestinal cells showed a low potential of TiO_2_ NPs to be transported across the intestinal epithelium [23,24]. However, contradicting results have been also reported, showing that TiO_2_ NPs could be internalized into cells and transported across the intestinal cell monolayers [25,26]. Meanwhile, several in vivo experiments demonstrated that TiO_2_ NPs could be absorbed into the body by different routes of exposure and distributed in various organs [27,28]. Moreover, TiO_2_ NPs were found to be taken up by Peyer’s Patch, which is present throughout the walls of the small intestine, after oral administration to rats [25]. It is worth noting that extremely low oral absorption efficiency of TiO_2_-NPs was demonstrated [25,28]. However, relatively little information is currently available regarding the relationships between the biological interactions of TiO_2_ NPs with biomatrices, oral absorption, and potential toxicity; furthermore, conflicting results have often been reported. 

In this study, we evaluated the biological behaviors of two kinds of TiO_2_ NPs, food grade (f-TiO_2_) and general grade (g-TiO_2_). Time-dependent physicochemical characterization of TiO_2_ NPs was performed in the presence of biomolecules. Albumin and glucose were chosen as representative model compounds as they are ubiquitous biomolecules and are often utilized as dispersing agents for various NPs [29,30]. Moreover, their biokinetics were evaluated in terms of oral absorption, tissue distribution, and excretion after a single-dose oral administration to rats. In order to address the possible intestinal transport mechanism of NPs, an in vitro model of human intestinal follicle-associated epithelium (FAE) established on a 3-dimensional (3D) culture system was utilized.

## 2. Results

### 2.1. Characterization

**Figure 1:**
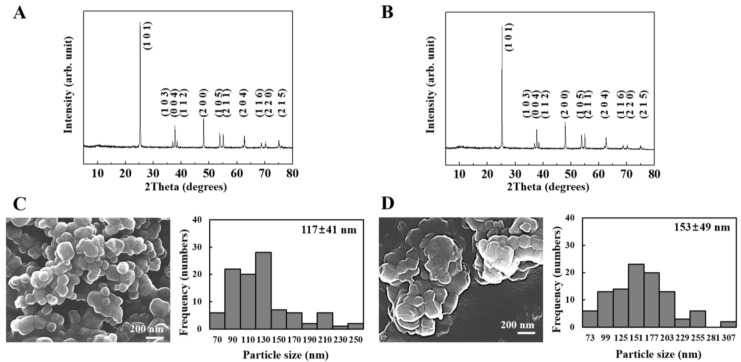
Powder X-ray diffraction patterns of (**A**) food grade TiO_2_ (f-TiO_2_) and (**B**) general grade TiO_2_ (g-TiO_2_). Scanning electron microscopic images and corresponding size distribution histograms of (**C**) f-TiO_2_ and (**D**) g-TiO_2_.

As shown in Figure 1A,B, two kinds of TiO_2_ NPs were determined to have an anatase (JCPDS No. 21-1272) structure, exhibiting characteristic diffraction peaks of (101), (004), (200), (105), (211) and (204) reflection planes. As the X-ray diffraction (XRD) patterns of the two different TiO_2_ NPs were similar in terms of intensity and sharpness, both NPs were considered to have a similar degree of crystallite size. Figure 1C,D displays scanning electron microscopic (SEM) images and the particle size distributions obtained from the SEM images. Both NPs had round, but irregular particle morphologies. Average primary particle sizes for 200 randomly selected particles in the SEM images were 117 ± 41 nm and 153 ± 49 nm for f-TiO_2_ and g-TiO_2_, respectively. In spite of the statistically similar primary particle size, g-TiO_2_ showed partial formation of agglomerates compared with f-TiO_2_. The Z-averages, based on the average hydrodynamic radii were 438 nm and 543 nm for f-TiO_2_ and g-TiO_2_, respectively; polydispersity indexes (PDI = [(standard deviation)/(average diameter)]^2^ obtained from hydrodynamic radius measurement), which reflect the homogeneity of a size distribution with a low value, were 0.398 and 0.479 for f-TiO_2_ and g-TiO_2_, respectively (Figure S1). These values imply that g-TiO_2_ tended to be more agglomerated than f-TiO_2_.

### 2.2. In Vitro and In Vivo Solubility

The in vitro solubility of TiO_2_ particles were evaluated in a simulated gastric fluid, because dissolution of NPs under acidic or physiological conditions can affect their bioavailability or biological fate, as in the case of ZnO and silver NPs [31,32,33]. The solubilities of f-TiO_2_ and g-TiO_2_ in simulated gastric fluid were 0.06% ± 0.03% and 0.05% ± 0.03%, respectively, which were fairly low and not significantly different from each other. In vivo solubilities in gastric fluid after a single-dose administration of 500 mg/kg via oral gavage to rats (the same dose used for the biokinetic study), were 0.05% ± 0.03% and 0.08% ± 0.05% for f-TiO_2_ and g-TiO_2_, respectively. The results reveal that both NPs, regardless of their primary particle size and degree of agglomeration, were dissolved less than 0.1% under acidic gastric condition.

### 2.3. Physicochemical Properties of TiO_2_ NPs in the Presence of Biomolecules

**Figure 2:**
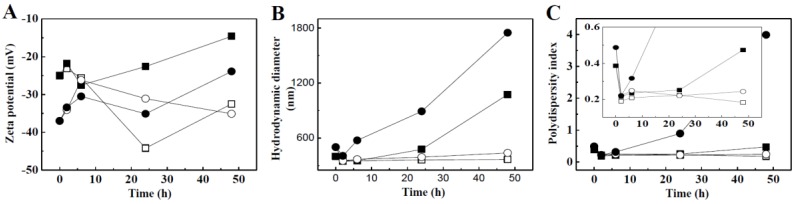
Time-dependent changes in colloidal properties. (**A**) Zeta potential; (**B**) hydrodynamic diameter; and (**C**) polydispersity index (PDI) values (PDI = [(standard deviation)/(average diameter)]^2^). Squares, f-TiO_2_; circles, g-TiO_2_; closed symbols, 1% glucose; open symbols, 1% albumin (□, f-TiO_2_ in 1% albumin; ■: f-TiO_2_ in 1% glucose; ○: g-TiO_2_ in 1% albumin; ●: g-TiO_2_ in 1% glucose). Data points at time 0 are the zeta potentials of each material in water without albumin or glucose.

Time-dependent changes in colloidal properties, such as zeta potential, hydrodynamic radius, and PDI values of the TiO_2_ NPs in the presence of albumin or glucose are displayed in Figure 2. f-TiO_2_ and g-TiO_2_ initially had average zeta potentials of −25 mV and −37 mV, respectively, but the initial negative zeta potentials shifted toward 0 upon adding 1% glucose. On the other hand, the values remained similar or shifted in the negative direction upon treatment with 1% albumin (Figure 2A). 

The hydrodynamic radii of the TiO_2_ NPs clearly showed different time-dependent changes according to the biomolecule type (Figure 2B). In the presence of 1% glucose, the hydrodynamic diameters of both types of TiO_2_ NPs gradually increased to reach more than 1000 nm (from 438 nm to 1075 nm and from 543 nm to 1749 nm for f-TiO_2_ and g-TiO_2_, respectively). However, 1% albumin reduced the hydrodynamic radii of both types of TiO_2_ NPs significantly (from 438 nm to 366 nm and from 543 nm to 437 nm for f-TiO_2_ and g-TiO_2_, respectively). Changes in PDI values were in accordance with hydrodynamic radius changes (Figure 2C). PDI values of both types of TiO_2_ NPs significantly reduced (from 0.385 to 0.190 and from 0.487 to 0.22 for f-TiO_2_ and g-TiO_2_, respectively) and were maintained below 0.25 in the presence of 1% albumin, showing fairly homogeneous distribution; however, 1% glucose gradually and dramatically increased PDI values upon incubation time, reaching 0.479 and 4.012 at 48 h for f-TiO_2_ and g-TiO_2_, respectively, suggesting the formation of large agglomerates. The degree of agglomeration was more remarked in g-TiO_2_ than in f-TiO_2_.

**Figure 3:**
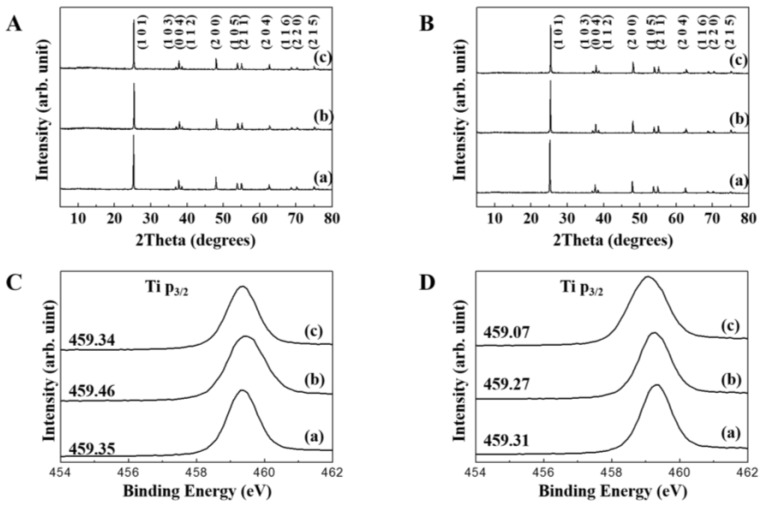
Powder X-ray diffraction patterns of (**A**) f-TiO_2_ and (**B**) g-TiO_2_ with or without biomolecules. X-ray photoelectron spectra of (**C**) f-TiO_2_ and (**D**) g-TiO_2_ with or without biomolecules. (a) TiO_2_ alone; (b) in 1% albumin; and (c) in 1% glucose.

In order to evaluate the changes in crystalline phase and surface chemistry of the TiO_2_ NPs in the presence of biomolecules, XRD and X-ray photoelectron spectra (XPS) of the NPs before and after biomolecule treatment were compared. As shown in Figure 3A,B, neither evolution of impurity phase nor significant changes in peak shape and intensity were found. These results reveal that both albumin and glucose did not affect dissolution of crystallite and phase transformation. According to quantitative analyses, less than 0.9% (*w*/*w*) of both albumin and glucose formed complexation with TiO_2_ NPs (data not shown), indicating only the surface of the NPs was covered with biomolecules. The surface interaction between NPs and biomolecules was further investigated with XPS (Figure 3C,D). The original binding energy of ~459.3 eV (Ti p_3/2_) slightly increased for f-TiO_2_ in the presence of albumin, whereas the binding energy for g-TiO_2_ reduced in the presence of either albumin or glucose. This kind of binding energy change is thought to result from the coordination bond formation between surface Ti(IV) and glucose or albumin, as previously reported in other NPs [34,35].

### 2.4. Intestinal Transport Mechanism

**Figure 4:**
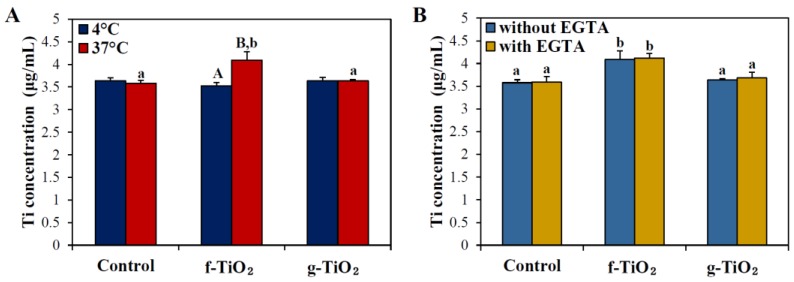
Intestinal transport mechanism of TiO_2_ NPs in vitro in a human follicle-associated epithelium (FAE) model. The transcytosis mechanism of particle transport was assessed by comparing transported amounts at 4 °C and 37 °C (**A**) and in the presence or absence of ethylene glycol tetraacetic acid (EGTA) at 37 °C (**B**). Mean values with different superscripts (A,B) for the same type of NP indicate significant differences at 4 °C and 37 °C (*p* < 0.05). Mean values with different superscripts (a,b) in the same figure indicate significant differences among the control (cells in medium), f-TiO_2_-, and g-TiO_2_-treated groups (*p* < 0.05).

The transport mechanism of two different types of TiO_2_ NPs across the intestinal epithelium was evaluated using an in vitro 3D cell culture system of a FAE model, by co-culturing human intestinal epithelial Caco-2 cells and human Raji B lymphocytes. This model represents specialized microfold (M) cells found in the FAE covering Peyer’s patches, which play a role in the transport of a wide range of materials, including microorganisms, macromolecules, and particles, as well as the intestinal immune system [36,37]. Figure 4A shows that only f-TiO_2_ was found to be transported by M cells and its elevated transport amount was detected only at 37 °C, not at 4 °C, indicating an energy-dependent transport pathway. In the Caco-2 monoculture system without Raji B cells, which represents intestinal tight junctions, no increase in the transport of either f-TiO_2_ or g-TiO_2_ was observed (data not shown). The transcytosis mechanism of particle translocation by M cells was further assessed by ethylene glycol tetraacetic acid (EGTA) pre-treatment, which induces the opening of enterocyte tight junctions. The transport of both types of TiO_2_ NPs was not affected by the EGTA treatment (Figure 4B).

### 2.5. Effects of Biomolecules on Oral Absorption

**Figure 5:**
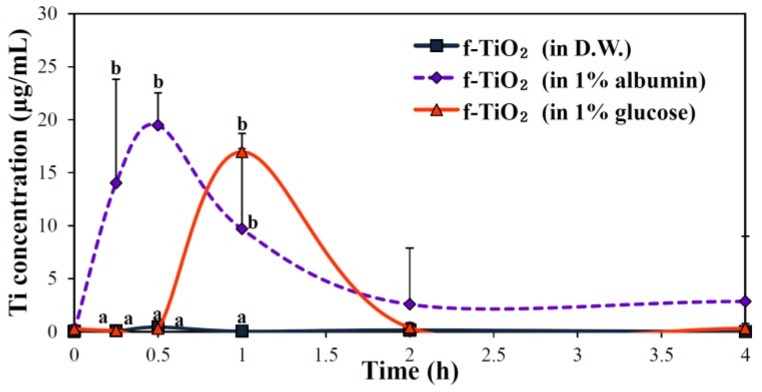
Effect of the presence of albumin or glucose on the plasma concentration-time profiles of f-TiO_2_ NPs after orally administering a single-dose (500 mg/kg) to rats. D.W., distilled water.

Oral absorption of f-TiO_2_ was investigated in the presence of albumin or glucose, after a single-dose administration to rats, in order to determine the effects of interactions between the NPs and biomolecules on the oral absorption of NPs. Figure 5 demonstrates that the plasma concentration-time profiles of f-TiO_2_ remarkably increased in the presence of albumin or glucose, showing peak concentration at 0.5 h and 1 h for albumin and glucose, respectively. Interestingly, the oral absorption of NPs considerably and significantly decreased when they were dispersed in distilled water (D.W.), showing ~0.01% of absorption (Table 1). It is worth noting that the absorption rate and efficiency of f-TiO_2_ was significantly greater in albumin than in glucose.

### 2.6. Effect of TiO_2_ NP Type on Oral Absorption

**Figure 6:**
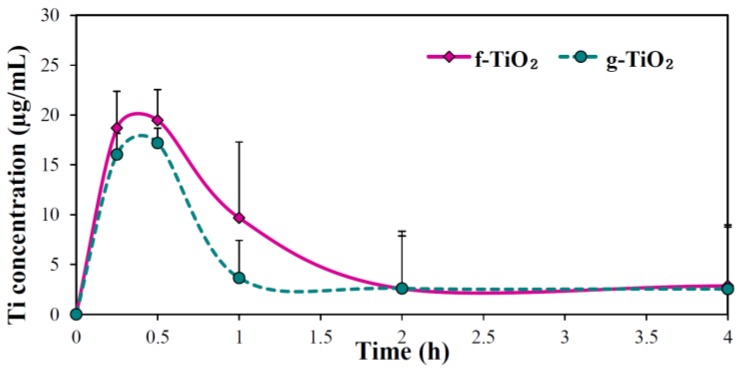
Plasma concentration-time profiles of the two kinds of TiO_2_ NPs after orally administering a single-dose (500 mg/kg) to rats.

The biokinetics of the two different types of TiO_2_ NPs were evaluated in the presence of 1% albumin because Figure 5 and Table 1 demonstrated that the oral absorption efficiency of NPs dispersed in albumin was higher than in glucose. Figure 6 shows similar plasma concentration versus time curves for f-TiO_2_ and g-TiO_2_ with a maximum concentration at 0.5 h post-administration. However, when the biokinetic parameters were calculated, *C*_max_, area under the plasma concentration-time curve (AUC), and absorption values between f-TiO_2_ and g-TiO_2_ were significantly different, while both NPs had similar *T*_max_, *T*_1/2_, and mean residence time (MRT) values (Table 2). Interestingly, total oral absorptions were low at <0.8% for both TiO_2_ NPs.

### 2.7. Tissue Distribution and Excretion Kinetics

**Figure 7:**
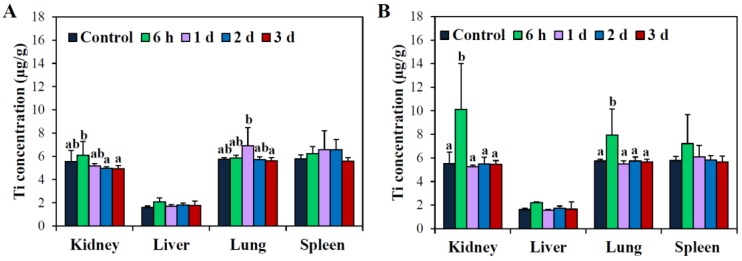
Tissue distribution of (**A**) f-TiO_2_ and (**B**) g-TiO_2_ after orally administering a single-dose (500 mg/kg) to rats. Mean values with different superscripts (a,b) in the same organs indicate significant differences between control and particle-treated animals (*p* < 0.05).

The biodistribution of the two different types of TiO_2_ NPs was assessed in possible target organs for accumulation [26,38], that is, kidneys, liver, lungs, and spleen following a single oral administration (500 mg/kg, the same dose used for biokinetic study) to rats (Figure 7). The f-TiO_2_ showed elevated Ti concentrations in the kidneys and lungs at 6 h and 1 day post-administration, respectively (Figure 7A); similarly, Ti levels in the kidneys and lungs of rats administered g-TiO_2_ significantly increased at 6 h. (Figure 7B). In both cases, elevated Ti levels decreased after 1 day post-administration in all organs analyzed. 

**Figure 8:**
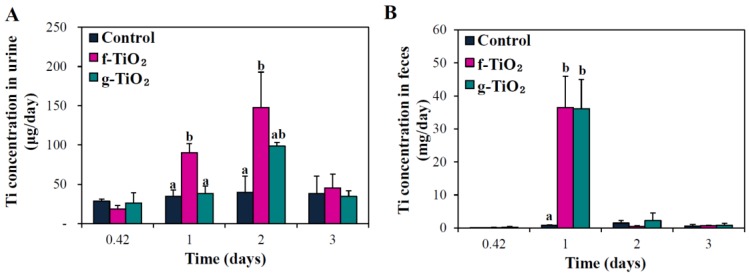
Excretion kinetics of f-TiO_2_ and g-TiO_2_ via (**A**) urine and (**B**) feces after orally administering a single-dose (500 mg/kg) to rats. Mean values with different superscripts (a,b) at the same time points indicate significant differences between the control and particle-treated rats (*p* < 0.05).

The excretion kinetics of the two different types of TiO_2_ NPs following oral administration to rats (500 mg/kg) were evaluated by measuring total Ti concentrations in urine and feces (Figure 8). Elevated Ti concentrations in urine were detected at 1–2 days post-administration and returned to normal levels thereafter (Figure 8A). Relatively rapid excretion kinetics through the feces was observed for both types of TiO_2_ NPs, showing increased Ti levels only at 1 day post-administration (Figure 8B). Table 3 summarizes the total excretion values of the TiO_2_ NPs and showed that more than 85% of the NPs were directly excreted in feces, but only a small portion of the NPs was eliminated in urine. An effect of NP type on excretion kinetics was not found.

## 3. Discussion

The biological responses of food and general grade TiO_2_ NPs were investigated based on their interactions with biomolecules, intestinal transport mechanism, and biokinetics. The physicochemical properties of f-TiO_2_ and g-TiO_2_ were found to be affected by the presence of glucose or albumin at 1% (*w*/*v*). Initial negative zeta potential values of TiO_2_ NPs turned more negative in the presence of albumin, while the zeta potential of NPs in glucose shifted toward 0 mV (Figure 2A). Zeta potential is often related to the colloidal stability of NPs; absolute zeta potential values higher than 30 mV are known to increase colloidal stability due to the strong charge-charge repulsion among NPs [39]. In this regard, albumin increased the colloidal stability of the NPs, while glucose resulted in the agglomeration of TiO_2_ particles. The hydrodynamic radii and PDI values of the TiO_2_ NPs reduced in the presence of albumin, but significantly increased with glucose (Figure 2B,C). A similar result was also reported by Šimundić et al., demonstrating the agglomeration tendency of TiO_2_ NPs in the presence of glucose [40]. These results suggest that albumin acts as a dispersant for TiO_2_ NPs. The dispersing effect of albumin was slightly higher for f-TiO_2_ than g-TiO_2_, and agglomeration by glucose was more significant for g-TiO_2_. This might be due to the slight difference in primary particle size and the formation of agglomerates in g-TiO_2_ (Figure S1D).

A stable suspension of TiO_2_ NPs in albumin, but agglomeration in glucose is likely to be highly related to the zeta potential change; however, agglomeration is not a simple consequence of a zeta potential value shift to zero. Agglomeration or stabilization of the TiO_2_ suspension could be induced by changes in surface chemistry, including surface interactions between biomolecules and TiO_2_ NPs. Glucose with negative charge centers at the hydroxyl groups cannot be easily adsorbed on the surfaces of negatively charged TiO_2_ NPs. Instead, they can be coordinated to the NP surfaces via interaction between surface Ti^4+^ and hydroxyls. The strength of coordination can be predicted by the hard-soft-acid-base theory, which explains the preferred combination of hard-hard or soft-soft species [41]. According to the theory, Ti^4+^ and hydroxyl in glucose are classified as hard acid and hard base, respectively, suggesting the possible bond formation between them. The XPS peak representing the binding energy for the metal cation shifted to lower energy when there was a coordination bond. In other words, coordinated electrons shield p_3/2_ electrons from nuclear charge [41], consequently resulting in binding energy reduction [42]. Both f-TiO_2_ and g-TiO_2_ showed a slight decrease in binding energy in the presence of albumin (Figure 3C,D). This may be attributed to the adsorption of albumin on the TiO_2_ surface through electrostatic interaction. Significant binding energy reduction of g-TiO_2_ in the presence of glucose may be attributed to the surface coordinated glucose (Figure 3D). Hence, it is likely that there was possible coordination bond between the TiO_2_ NPs and glucose, which made NP-glucose networks, finally leading to agglomeration. On the other hand, albumin is thought to attach to the NP surface through relatively weak van der Waals interactions, contributing to a relatively stable NP dispersion. The zeta potential change in Figure 2 could be explained by different surface interactions between albumin or glucose and TiO_2_-NPs.

When the human body is exposed to NPs by the oral route, the question as to whether NPs can be transported across the intestinal epithelium is fundamental in understanding their potential toxicity and absorption efficiency. Most studies have demonstrated little or low transportation of NPs in intestinal monocultures of Caco-2 cells [9,23,43]. However, this model represents enterocyte monolayers with a dense network of tight junctions, thereby preventing NP translocation. In the present study, we used a 3D co-culture system of Caco-2 cells and Raji B cells, a FAE model, to investigate NP transport by M cells. Indeed, M cells found in the FAE have a high capacity of transcytosis and can transport various materials, including NPs, soluble tracers, bacteria, and viruses [37]. Moreover, M cells are also known to be capable of energy-dependent endocytosis [16,24,36,38]. In the present study, f-TiO_2_ was found to be transported by M cells in an energy-dependent manner, and exhibited significantly increased transport only when the temperature increased to 37 °C (Figure 4A), which was not in the Caco-2 monolayer system (data not shown). On the other hand, the transport of f-TiO_2_ was not influenced by EGTA treatment, which suggests that NPs were efficiently transcytosed by M cells and a paracellular transport mechanism does not play a major role in NP uptake. No significantly increased transport of g-TiO_2_ may be related to their agglomerated fate in biological media (Figure 2B,C). It seems that the slightly increased transport of f-TiO_2_ by M cells is related to its low oral absorption (Table 2). Meanwhile, no significantly increased transport of g-TiO_2_ may be attributed to its more agglomerated fate under physiological conditions than f-TiO_2_. Brun et al. demonstrated that TiO_2_ NPs accumulated and translocated into M cells by using a FAE model and TEM image analysis [26], and this is consistent with our results. 

The effects of the presence of albumin or glucose on the biokinetics of TiO_2_ NPs were evaluated, which also aimed to identify an appropriate dispersant for in vivo administration. As shown in Figure 5 and Table 1, f-TiO_2_ dispersed in albumin or glucose entered the bloodstream more rapidly and highly as compared with the NP dispersion in D.W. Moreover, f-TiO_2_ in albumin had the highest absorption efficiency and rapid absorption rate than in glucose. This result implies that interactions between TiO_2_ and biomolecules could affect biokinetics and that these interactions are highly dependent on the physicochemical properties of the NPs and biomolecules. As a consequence, it is likely that albumin serves as a dispersant for TiO_2_, which is in good agreement with colloidal behaviors (Figure 2). 

Based on the above result, the biokinetics of f-TiO_2_ and g-TiO_2_ dispersed in albumin were carried out to determine the effects of NP type (Figure 6). Oral absorption efficiency was statistically greater for f-TiO_2_ than g-TiO_2_ (Table 2), but their biokinetic profiles were similar, possibly because of the similar primary particle size (Figure 1). Oral absorption of f-TiO_2_ was about 1.5 fold higher than g-TiO_2_, but the total absorption amounts of both NPs were less than 0.8% (Table 2), suggesting extremely low oral absorption efficiency. In one study, TiO_2_ NPs were found to have low absorption even after oral administration for 13 weeks, showing Ti levels at 0.4 to 0.5 µg/g in whole blood [26]. Therefore, it is likely that only a small portion of TiO_2_ NPs could be absorbed into the body. The significantly enhanced oral absorption of f-TiO_2_ compared to g-TiO_2_ can be explained by their efficient intestinal transport mechanism by M cells (Figure 4), which can be also associated with relatively small hydrodynamic radii in aqueous solution (Figure S1). Meanwhile, the biological fate of both types of TiO_2_ NPs seems to be a particulate fate, based on their extremely low in vivo dissolution properties (~0.05%). Particulate persistence of TiO_2_ NPs in gut epithelium or in Peyer’s patches was also suggested by Janer et al. and Brun et al. [25,26].

There was no significant difference in tissue distribution patterns (Figure 7) or excretion kinetics (Figure 8) depending on TiO_2_ type. Both types of TiO_2_ NPs accumulated only slightly in the kidneys and lungs at 6–24 h post-administration. Cho et al. reported that no significant increase in Ti levels occurred in sampled organs, including brain, liver, kidneys, and spleen, after 13-week repeated oral administration of TiO_2_ NPs dispersed in D.W. [28]. On the other hand, the elevated levels of all particles detected in the kidneys also indicate possible renal excretion, as evidenced in Figure 8A. However, fecal excretion seems to play a major role in NP elimination, which is in good agreement with the previous report [14]. Taken together, most TiO_2_ NPs were not absorbed, and were directly eliminated through the feces. 

## 4. Materials and Methods

### 4.1. Materials and Characterization

Food grade TiO_2_ NP (f-TiO_2_) and general grade TiO_2_ NP (g-TiO_2_) were purchased from Avantor Performance Materials Inc. (Center Valley, PA, USA) and Alfa Aesar Johnson Matthey Co. (Karlsruhe, Germany), respectively. Glucose and albumin were purchased from Sigma-Aldrich (St. Louis, MO, USA). Powder X-ray diffraction (XRD) patterns for NPs were measured using a X-ray diffractometer (D2phaser, Bruker AXS Inc., Madison, WI, USA) with Ni-filtered CuKα radiation. Particle size and morphology were examined by scanning electon microscopy (SEM; FEI QUANTA 250 FEG, Hillsboro, OR, USA). Zeta potential and hydrodynamic radii of the NPs in aqueous suspension were measured with an ELSZ-1000 instrument (Otsuka, Osaka, Japan).

### 4.2. Solubility and ICP-AES Analysis

For the in vitro solubility test, both NPs (5 mg/mL) were dispersed in simulated gastric fluid (0.2% NaCl, 0.32% pepsin, pH 1.5) and phosphate buffered saline (PBS, pH 7.4). After different incubation times at 37 °C, supernatants were collected by ultracentrifugation for 15 min. The in vivo solubility of the particles was analyzed after a single dose oral administration of 500 mg/kg to male rats as previously reported [44]. Briefly, stomachs were collected at 15 min post-administration after CO_2_ euthanasia, rinsed with saline, and the gastric fluids were extracted. After centrifugation of the gastric fluids at 12,000× *g* for 3 min at 4 °C, supernatants were obtained and passed through a syringe filter (pore size 0.45 µm; Advantech, Taipei, Taiwan) and pre-digested with ultrapure nitric acid (HNO_3_) and hydrogen peroxide (H_2_O_2_). For the quantitative analysis of Ti, the plasma samples were digested overnight in 10 mL of sulfuric acid (H_2_SO_4_) solution and the remaining solution was removed by heating. Aqua regia solution (HCl:HNO_3_ = 3:1, 5 mL) was then added. Finally, after adding HNO_3_ and H_2_O_2_ solution, the samples were heated at 180 °C until the solution was colorless and clear. The solutions were finally diluted to 5 mL with 2% HNO_3_ and total Ti contents were determined by inductively compled plasma-atomic emission spectroscopy (ICP-AES; JY2000 Ultrace, HORIBA Jobin Yvon, Stow, MA, USA).

### 4.3. Physicochemical Properties of TiO_2_ NPs in the Presence of Biomolecules

In order to evaluate the physicochemical properties of TiO_2_ NPs in different biological environments, TiO_2_ NPs were dispersed in 1% (*w*/*v*) solution of either albumin or glucose. The zeta potential change of the NPs after dispersion in each solution was recorded at 0, 2, 6, 24 and 48 h with light scattered electrophoresis (ELSZ-1000, Otsuka, Osaka, Japan). Hydrodynamic radii and polydispersity indexes (PDI) with respect to time were measured with dynamic light scattering (DLS) using an ELSZ-1000. The surface interaction between particles and biological molecules was investigated by measuring the binding energy of Ti p_3/2_ electrons, utilizing a X-ray photoelectron spectrophotometer (XPS; Thermo Fisher K-ALPHA, Waltham, MA, USA).

### 4.4. Intestinal Transport Mechanism

An in vitro model of human intestinal FAE, representing M cells, was prepared according to the protocol developed by des Rieux et al. [45]. Human intestinal epithelial Caco-2 cells were purchased from the Korean Cell Line Bank (Seoul, Korea) and grown in Dulbecco’s Modified Eagle Medium (DMEM) supplemented with 10% fetal bovine serum, 1% non-essential amino acids, 1% l-glutamine, 100 units/mL penicillin, and 100 μg/mL streptomycin under the standard condition as described elsewhere [46]. Briefly, Transwell^®^ polycarbonate inserts (Corning Costar, New York, NY, USA) were coated with Matrigel^TM^ basement membrane matrix (Becton Dicknson, Bedford, MA, USA), prepared in pure DMEM, and were then placed at room temperature for 1 h. The supernatants were removed and the inserts were washed with DMEM. Caco-2 cells (5 × 10^5^ cells) were grown on the upper insert side and incubated for 14 days. Then, non-adherent human Burkitt’s lymphoma Raji B cells (5 × 10^5^ cells, Korean Cell Line Bank) in the same medium were added to the basolateral insert compartment, and the co-cultures were maintained for 5 days. TiO_2_ NPs (250 μg/mL) were prepared in Hank’s Balanced Salt Solution (HBSS, pH 7.4) buffer, and the apical medium of the cell monolayers was replaced by a particle suspension and incubated for 6 h. Basolateral solutions were then sampled and the concentrations of transported particles were estimated by measuring total Ti levels as described in the “Solubility and ICP-AES analysis’’.

To investigate the role of the energy-dependent mechanism, the same experiments were performed at 4 °C and 37 °C, respectively. Transcellular particle transport by M cells was also evaluated as follows; inserts were incubated apically and basolaterally with 2.5 mM EGTA in HBSS (pH 7.4) twice for 15 min at 37 °C. Fresh EGTA was then placed basolaterally and the particle suspension was added to the apical side of the cell monolayers, and incubation was continued for 6 h. Then, the basolateral solution was sampled and anlayzed by ICP-AES. The cytotoxicity in the presence of particles or EGTA was assessed by measuring lactate dehydrogenase (LDH) release from the cytoplasm of damaged cells in the apical medium. The experiment was repeated three times on three separate days.

### 4.5. Animals

Five-week old male Sprague Dawley (SD) rats weighing 130–150 g were purchased from Nara Biotech Co., Ltd. (Seoul, Korea). Animals were housed in plastic laboratory animal cages in a ventilated room, which was maintained at 20 ± 2 °C/60% ± 10% relative humidity with a 12 h light/dark cycle. Water and commercial laboratory complete food for rats were available ad libitum. Animals were environmentally acclimated 7 days before treatment. All animal experiments were performed in compliance with the guidelines issued by the Animal and Ethics Review Committee of Seoul Women’s University, Seoul, Korea.

### 4.6. Biokinetic Study

Six male rats per group were administered a single-dose of 500 mg/kg TiO_2_ NPs, dispersed for 48 h in D.W., 1% (*w/v*) glucose or 1% (*w/v*) albumin, via oral gavage, and the blood samples were collected via the tail vein at several time points (0, 0.5, 1, 2, 3, 4, 6 and 10 h) after oral administration. The highest dose at which acute toxicity was not observed was chosen based on the pre-toxicity test of each particle. Body weight changes, behaviors, and symptoms were recorded daily after treatment. An additional group of six rats received an equivalent volume of 1% (*w/v*) glucose or 1% (*w/v*) albumin solution and were used as controls. The blood samples were centrifuged at 3000 rpm for 15 min at 4 °C to obtain the plasma. The following biokinetic parameters were estimated using Kinetica software (version 4.4, Thermo Fisher Scientific, Waltham, MA, USA): Maximum concentration (*C*_max_), time to maximum concentration (*T*_max_), area under the plasma concentration-time curve (AUC), half-life (*T*_1/2_), and mean residence time (MRT). 

For tissue distribution and excretion kinetic studies, 500 mg/kg TiO_2_ NPs in 5% albumin were orally administered. Tissue samples of the kidneys, liver, lungs, and spleen were collected at several time points (6 h, and 1, 2 and 3 days) following CO_2_ euthanasia. Urine and feces were also collected at 6 h, and 1, 2, and 3 days post-administration. 

### 4.7. Statistical Analysis

Results were expressed as means ± standard deviations. A one-way analysis of variance (ANOVA) with Tukey’s Test was performed using SAS software, version 9.4 (SAS Institute Inc., Cary, NC, USA) to determine the significance of differences between the experimental groups. Statistical significance was accepted for *p* < 0.05.

## 5. Conclusions

In conclusion, interactions between TiO_2_ NPs and biomolecules can highly affect oral absorption of NPs, as demonstrated by the higher absorptions of f-TiO_2_ in albumin or glucose than in D.W., which is associated with their colloidal behaviors being affected by surface characteristics. Therefore, it seems that it is essential to consider the interactions between NPs and biomolecules for toxicity and efficacy studies. The oral absorption of TiO_2_ NPs is likely to be primarily associated with their efficient intestinal transport by M cells and can be affected by particle type. However, overall absorptions of f-TiO_2_ and g-TiO_2_ were less than 0.8%, and most of the particles were directly eliminated through the feces, suggesting low toxicity potential of TiO_2_ NPs.

## Figures and Tables

**Table 1:** Biokinetic parameters and oral absorption of food grade TiO_2_ (f-TiO_2_) particles in different biomolecules after a single oral administration to rats.

	f-TiO_2_ (in D.W.)	f-TiO_2_ (in 1% Albumin)	f-TiO_2_ (in 1% Glucose)
***C*_max _(mg/L)**	0.46 ± 0.09 ^a^	20.02 ± 1.35 ^c^	16.96 ± 1.110 ^b^
***T*_max _(h)**	0.5 ^a^	0.5 ^a^	1 ^b^
**AUC (h × mg/L)**	0.54 ± 0.03 ^a^	30.45 ± 8.34 ^c^	11.59 ± 1.42 ^b^
***T*_1/2_**	0.93 ± 0.18 ^a^	1.11 ± 0.09 ^a^	1.36 ± 0.11 ^b^
**MRT (h)**	1.63 ± 0.30 ^a^	1.58 ± 0.48 ^a^	1.70 ± 0.07 ^a^
**Absorption (%)**	0.01 ± 0.00 ^a^	0.78 ± 0.21 ^c^	0.30 ± 0.04 ^b^

Mean values with different superscripts (a–c) in the same column are significantly different (*p *< 0.05). Absorption (%) was calculated based on AUC values. Abbreviation: D.W., distilled water; *C*_max_, maximum concentration; *T*_max_, time to maximum concentration; AUC, area under the plasma concentration-time curve; *T*_1/2_, half-life; MRT, mean residence time.

**Table 2:** Biokinetic parameters and oral absorption of different types of TiO_2_ particles after a single oral administration to rats.

	f-TiO_2_	g-TiO_2_
***C*_max_ (mg/L)**	20.02 ± 1.35 ^a^	17.66 ± 0.98 ^b^
***T*_max_ (h)**	0.5 ^a^	0.5 ^a^
**AUC (h × mg/L)**	30.45 ± 8.34 ^a^	20.28 ± 6.7 ^b^
***T*_1/2_**	1.11 ± 0.09 ^a^	1.29 ± 0.49 ^a^
**MRT (h)**	1.58 ± 0.48 ^a^	1.80 ± 0.71 ^a^
**Absorption (%)**	0.78 ± 0.21 ^a^	0.52 ± 0.17 ^b^

Mean values with different superscripts (a,b) in the same column are significantly different (*p* < 0.05). Absorption (%) was calculated based on AUC values.

**Table 3:** Excretion profiles of the different types of TiO_2_ particles after a single oral administration to rats.

	f-TiO_2_	g-TiO_2_
**Excretion via urine (%)**	0.31 ± 0.15	0.16 ± 0.02
**Excretion via feces (%)**	85.20 ± 18.70	86.48 ± 16.96

## Supplementary Materials

The following are available online at http://www.mdpi.com/2079-4991/6/12/225/s1.

## References

[1] Berger C., Song Z., Li T., Li X., Ogbazghi A.Y., Feng R., Dai Z., Marchenkov A.N., Conrad E.H., First P.N. (2004). Ultrathin epitaxial graphite: 2D electron gas properties and a route toward graphene-based nanoelectronics. J. Phys. Chem. B.

[2] Zhang J., Teo J., Chen X., Asakura H., Tanaka T., Teramura K., Yan N. (2014). A series of NiM (M = Ru, Rh, and Pd) bimetallic catalysts for effective lignin hydrogenolysis in water. ACS Catal..

[3] Peer D., Karp J.M., Hong S., Farokhzad O.C., Margalit R., Langer R. (2007). Nanocarriers as an emerging platform for cancer therapy. Nat. Nanotechnol..

[4] Kashyap P.L., Xiang X., Heiden P. (2015). Chitosan nanoparticle based delivery systems for sustainable agriculture. Int. J. Biol. Macromol..

[5] Buso D., Pacifico J., Martucci A., Mulvaney P. (2007). Gold-nanoparticle-doped TiO_2_ semiconductor thin films: Optical characterization. Adv. Funct. Mater..

[6] Jagadale T.C., Takale S.P., Sonawane R.S., Joshi H.M., Patil S.I., Kale B.B., Ogale S.B. (2008). N-doped TiO_2_ nanoparticle based visible light photocatalyst by modified peroxide sol-gel method. J. Phys. Chem. C.

[7] Bodurov I., Yovcheva T., Sainov S. (2014). PMMA films refractive index modulation via TiO_2_ nanoparticle inclusions and corona poling. Colloid Polym. Sci..

[8] Weir A., Westerhoff P., Fabricius L., Hristovski K., von Goetz N. (2012). Titanium dioxide nanoparticles in food and personal care products. Environ. Sci. Technol..

[9] Peters R.J.B., van Bemmel G., Herrera-Rivera Z., Helsper H.P.F.G., Marvin H.J.P., Weigel S., Tromp P.C., Oomen A.G., Rietveld A.G., Bouwmeester H. (2014). Characterization of titanium dioxide nanoparticles in food products: Analytical methods to define nanoparticles. J. Agric. Food Chem..

[10] Popov A.P., Kirillin M.Y., Priezzhev A.V., Lademann J., Hast J., Myllyla R. Optical Sensing of Titanium Dioxide Nanoparticles within Horny Layer of Human Skin and Their Protecting Effect against Solar UV Radiation. Proceedings of the SPIE-The International Society for Optical Engineering.

[11] Donaldson K., Beswick P.H., Gilmour P.S. (1996). Free radical activity associated with the surface of particles: A unifying factor in determining biological activity?. Toxicol. Lett..

[12] Uchino T., Tokunaga H., Ando M., Utsumi H. (2002). Quantitative determination of OH radical generation and its cytotoxicity induced by TiO_2_–UVA treatment. Toxicol. In Vitro.

[13] Zhang A.-P., Sun Y.-P. (2004). Photocatalytic killing effect of TiO_2_ nanoparticles on Ls-174-t human colon carcinoma cells. World J. Gastroenterol..

[14] MacNicoll A., Kelly M., Aksoy H., Kramer E., Bouwmeester H., Chaudhry Q. (2015). A study of the uptake and biodistribution of nano-titanium dioxide using in vitro and in vivo models of oral intake. J. Nanopart. Res..

[15] Warheit D.B., Hoke R.A., Finlay C., Donner E.M., Reed K.L., Sayes C.M. (2007). Development of a base set of toxicity tests using ultrafine TiO_2_ particles as a component of nanoparticle risk management. Toxicol. Lett..

[16] Warheit D.B., Brown S.C., Donner E.M. (2015). Acute and subchronic oral toxicity studies in rats with nanoscale and pigment grade titanium dioxide particles. Food Chem. Toxicol..

[17] Wang C., Xie Y., Li A., Shen H., Wu D., Qiu D. (2014). Bioactive nanoparticle through postmodification of colloidal silica. ACS Appl. Mater. Interfaces.

[18] Lee S.-Y., Harris M.T. (2006). Surface modification of magnetic nanoparticles capped by oleic acids: Characterization and colloidal stability in polar solvents. J. Colloid Interface Sci..

[19] Nooney R.I., White A., O’Mahony C., O’Connell C., Kelleher S.M., Daniels S., McDonagh C. (2015). Investigating the colloidal stability of fluorescent silica nanoparticles under isotonic conditions for biomedical applications. J. Colloid Interface Sci..

[20] Takahashi M., Yoshino T., Matsunaga T. (2010). Surface modification of magnetic nanoparticles using asparagines-serine polypeptide designed to control interactions with cell surfaces. Biomaterials.

[21] Oh N., Park J.-H. (2014). Surface chemistry of gold nanoparticles mediates their exocytosis in macrophages. ACS Nano.

[22] Lynch I., Salvati A., Dawson K.A. (2009). Protein-nanoparticle interactions: What does the cell see?. Nat. Nanotechnol..

[23] Yang Y.-X., Song Z.M., Cheng B., Xiang K., Chen X.-X., Liu J.-H., Cao A., Wang Y., Liu Y., Wang H. (2014). Evaluation of the toxicity of food additive silica nanoparticles on gastrointestinal cells. J. Appl. Toxicol..

[24] Song Z.M., Chen N., Liu J.-H., Tang H., Deng X., Xi W.-S., Han K., Cao A., Liu Y., Wang H. (2015). Biological effect of food additive titanium dioxide nanoparticles on intestine: An in vitro study. J. Appl. Toxicol..

[25] Janer G., del Molino E.M., Fernández-Rosas E., Fernández A., Vázquez-Campos S. (2014). Cell uptake and oral absorption of titanium dioxide nanoparticles. Toxicol. Lett..

[26] Brun E., Barreau F., Veronesi G., Fayard B., Sorieul S., Chanéac C., Carapito C., Rabilloud T., Mabondzo A., Herlin-Boime N. (2014). Titanium dioxide nanoparticle impact and translocation through ex vivo, in vivo and in vitro gut epithelia. Part. Fibre Toxicol..

[27] Wang J., Zhou G., Chen C., Yu H., Wang T., Ma Y., Jia G., Gao Y., Li B., Sun J. (2007). Acute toxicity and biodistribution of different sized titanium dioxide particles in mice after oral administration. Toxicol. Lett..

[28] Cho W.-S., Kang B.-C., Lee J.K., Jeong J., Che J.-H., Seok S.H. (2013). Comparative absorption, distribution, and excretion of titanium dioxide and zinc oxide nanoparticles after repeated oral administration. Part. Fibre Toxicol..

[29] Schilling C.H., Sikora M., Tomasik P., Li C., Garcia V. (2002). Rheology of alumina–nanoparticle suspensions: Effects of lower saccharides and sugar alcohols. J. Eur. Ceram. Soc..

[30] Valenti L.E., Giacomelli C.E. (2015). Unaffected features of BSA stabilized Ag nanoparticles after storage and reconstitution in biological relevant media. Colloids Surf. B.

[31] Morris V.J. (2011). Emerging roles of engineered nanomaterials in the food industry. Trends Biotechnol..

[32] Rashidi L., Khosravi-Darani K. (2011). The applications of nanotechnology in food industry. Crit. Rev. Food Sci..

[33] Dekkers S., Krystek P., Peters R.J.B., Lankveld D.P.K., Bokkers B.G.H., van Hoeven-Arentzen P.H., Bouwmeester H., Oomen A.G. (2011). Presence and risks of nanosilica in food products. Nanotoxicology.

[34] Kim K.-M., Kim H.M., Lee W.J., Lee C.-W., Kim T.-I., Lee J.-K., Jeong J., Paek S.-M., Oh J.-M. (2014). Surface treatment of silica nanoparticles for stable and charge-controlled colloidal silica. Int. J. Nanomed..

[35] Kim K.-M., Choi M.-H., Lee J.-K., Jeong J., Kim Y.-R., Kim M.-K., Paek S.-M., Oh J.-M. (2014). Physicochemical properties of surface charge-modified ZnO nanoparticles with different particle sizes. Int. J. Nanomed..

[36] Joint FAO/WHO Expert Committee on Food Additives (2006). Combined Compendium of Food Additive Specifications.

[37] European Commission (1994). European parliament and council directive 94/36/EC of 30 June 1994 on colours for use in foodstuffs. Off. J. Eur. Communities.

[38] Jovanović B., Cvetković V.J., Mitrović T.L. (2016). Effects of human food grade titanium dioxide nanoparticle dietary exposure on Drosophila melanogaster survival, fecundity, pupation and expression of antioxidant genes. Chemosphere.

[39] Hanaor D., Michelazzi M., Leonelli C., Sorrell C.C. (2012). The effects of carboxylic acids on the aqueous dispersion and electrophoretic deposition of ZrO_2_. J. Eur. Ceram. Soc..

[40] Šimundić M., Barbara D., Vid Šuštar J.Z., Jernej Z., Roman Š., Darko M., Deniz E., Henry H., Damjana D., Veronika K.-I. (2013). Effect of engineered TiO_2_ and ZnO nanoparticles on erythrocytes, platelet-rich plasma and giant unilamelar phospholipid vesicles. BMC Vet. Res..

[41] Miessler G.L., Tarr D.A. (2003). Inorganic Chemistry.

[42] Kim K.-M., Kim H.-M., Choi M.-H., Lee J.K., Jeong J., Lee M.-H., Kim Y.S., Paek S.-M., Oh J.-M. (2014). Colloidal properties of surface coated colloidal silica nanoparticles in aqueous and physiological solutions. Sci. Adv. Mater..

[43] Westerhoff P., Song G., Hristovski K., Kiser M.A. (2011). Occurrence and removal of titanium at full scale wastewater treatment plants: Implications for TiO_2_ nanomaterials. J. Environ. Monit..

[44] Paek H.-J., Chung H.-E., Lee J.-A., Kim M.-K., Lee Y.-J., Kim M.-S., Kim S.-H., Maeng E.-H., Lee J.K., Jeong J. (2014). Quantitative determination of silica nanoparticles in biological matrices and their pharmacokinetics and toxicokinetics in rats. Sci. Adv. Mater..

[45] Des Rieux A., Fievez V., Théate I., Mast J., Préat V., Schneider Y.-J. (2007). An improved in vitro model of human intestinal follicle-associated epithelium to study nanoparticle transport by M cells. Eur. J. Pharm. Sci..

[46] Kim M.-K., Lee J.-A., Jo M.-R., Kim M.-K., Kim H.-M., Oh J.-M., Song N.W., Choi S.-J. (2015). Cytotoxicity, uptake behaviors, and oral absorption of food grade calcium carbonate nanomaterials. Nanomaterials.

