# Supplementary material for: Titanium Dioxide Nanoparticle-Biomolecule Interactions Influence Oral Absorption
Source: Nanomaterials (Basel). 2016 Nov 29;6(12):225. doi: 10.3390/nano6120225 (PMC5302714; doi:10.3390/nano6120225)
Supplement: Supplementary file 1 [file nanomaterials-06-00225-s001.pdf]

Supplementary Materials: Titanium Dioxide Nanoparticle-Biomolecule Interactions Influence Oral Absorption

Mi-Rae Jo, Jin Yu, Hyoung-Jun Kim, Jae-Ho Song, Kyoung-Min Kim, Jae-Min Oh and Soo-Jin Choi

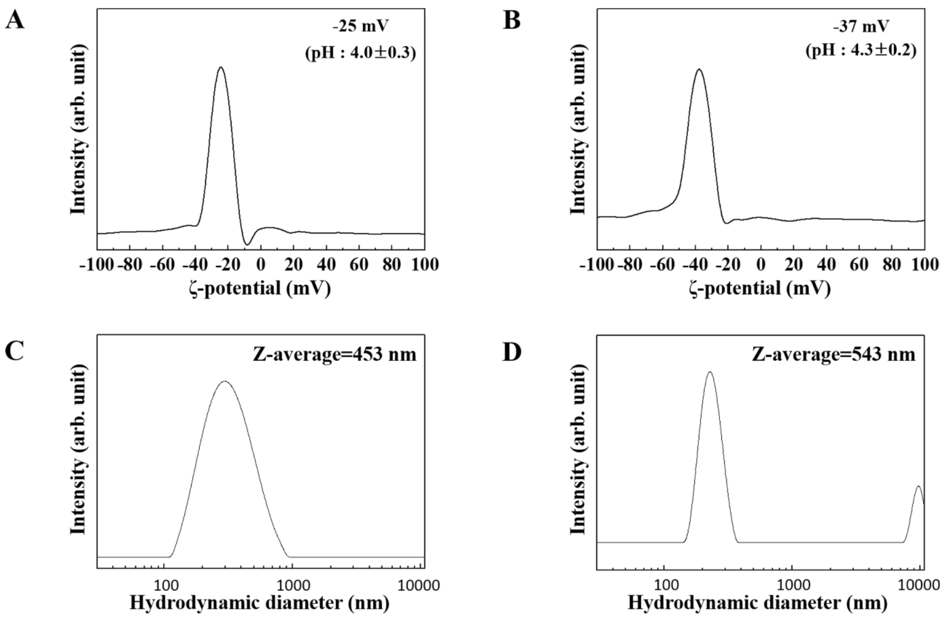

Figure S1. Zeta-potential value and hydrodynamic diameter TiO_2 without biomoleculs. (**A**,**C**) Food grade TiO_2; (**B**,**D**) General grade TiO_2.

© 2016 by the authors. Submitted for possible open access publication under the terms and conditions of the Creative Commons Attribution (CC-BY) license (http://creativecommons.org/licenses/by/4.0/).
